# Crystal structure of di­aqua­(3,14-diethyl-2,6,13,17-tetra­aza­tri­cyclo­[16.4.0.0^7,12^]docosa­ne)copper(II) (3,14-diethyl-2,6,13,17-tetra­aza­tri­cyclo[16.4.0.0^7,12^]docosa­ne)copper(II) tetra­bromide dihydrate, [Cu(C_22_H_44_N_4_)(H_2_O)_2_][Cu(C_22_H_44_N_4_)]Br_4_·2H_2_O

**DOI:** 10.1107/S205698902100551X

**Published:** 2021-05-28

**Authors:** Dohyun Moon, Sunghwan Jeon, Woo Taik Lim, Keon Sang Ryoo, Jong-Ha Choi

**Affiliations:** a Pohang Accelerator Laboratory, POSTECH, Pohang 37673, Republic of Korea; b Department of Chemistry, Andong National University, Andong 36729, Republic of Korea

**Keywords:** crystal structure, macrocycle, double copper(II) complex, bromide, hydrogen bonding, synchrotron radiation

## Abstract

The crystal structure of the new double Cu^II^ complex salt, [Cu(*L*)(H_2_O)_2_][Cu(*L*)]Br_4_·2H_2_O (*L* = 3,14-diethyl-2,6,13,17-tetra­aza­tri­cyclo­[16.4.0.0^7,12^]docosane, C_22_H_44_N_4_) has been determined using synchrotron radiation. The asymmetric unit contains one half of a [Cu(*L*)(H_2_O)_2_]^2+^ cation, one half of a [Cu(*L*)]^2+^ cation (both completed by crystallographic inversion symmetry), two bromide anions and one water solvent mol­ecule. The Cu^II^ atom in the first complex exists in a tetra­gonally distorted octa­hedral environment with the four N atoms of the macrocyclic ligand in equatorial and two aqua ligands in axial positions, whereas the Cu^II^ atom in the second complex exists in a square-planar environment defined by the four nitro­gen atoms of the macrocyclic ligand. The two macrocyclic rings adopt the most stable *trans*-III configuration with normal Cu—N bond lengths from 2.016 (3) to 2.055 (3) Å and an axial Cu—O bond length of 2.658 (4) Å. The crystal structure is stabilized by inter­molecular hydrogen bonds involving the macrocycle N—H or C—H groups and the O—H groups of water mol­ecules as donor groups, and the O atoms of water mol­ecules and bromide anions as acceptor groups, giving rise to a one-dimensional network extending parallel to [100].

## Abstract 2

In the title complex, [Cu(C_22_H_44_N_4_)(H_2_O)_2_][Cu(C_22_H_44_N_4_)]Br_4_·2H_2_O, each of the two complex cations lies about an inversion center. The two macrocyclic rings adopt the most stable *trans*-III configuration. In the crystal, O—H⋯Br, N—H⋯Br, N—H⋯O and C—H⋯O hydrogen bonds connect the complex cations, bromide anions, semi-coordinating H_2_O ligands and water solvent mol­ecules, forming a one-dimensional network extending parallel [100].

## Chemical context   

According to recent investigations, 1,4,8,11-tetra­aza­cyclo­tetra­decane (cyclam) derivatives and their transition-metal complexes show anti­viral, anti­microbial and anti­bacterial activities (Ronconi & Sadler, 2007 ▸; Ross *et al.*, 2012 ▸; Alves *et al.*, 2017 ▸, 2019 ▸; De Clercq, 2019 ▸). In particular, novel cyclams and their Cu^II^ and Fe^III^ complexes have been studied as anti­cancer agents (Pilon *et al.*, 2019 ▸). The design of new drugs with these moieties depends on the configuration, substituent and coordination behavior of the cyclam-based macrocycle (Valks *et al.*, 2006 ▸).

3,14-Diethyl-2,6,13,17-tetra­aza­tri­cyclo­(16.4.0.0^7,12^)doco­sane (C_22_H_44_N_4_, *L*) also contains a cyclam backbone with cyclo­hexane subunits and ethyl groups at the carbon atoms (Subhan & Choi, 2014 ▸). To the best of our knowledge, the preparation and crystal structure for any double metal complex containing the macrocycle *L* have not been reported.
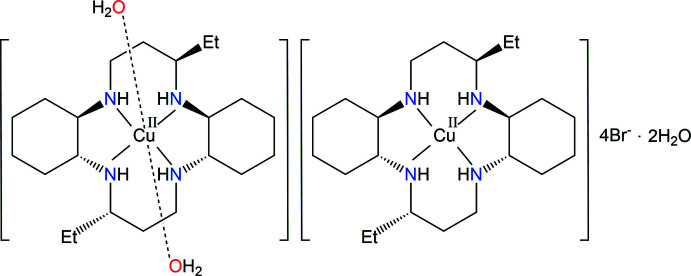


Here, we report on the synthesis and structural characterization of the new double Cu^II^ complex, namely, [Cu(*L*)(H_2_O)_2_][Cu(*L*)]Br_4_·2H_2_O, (I), to determine the configuration of the macrocycles and the bonding properties of the water mol­ecules and bromide anions in the crystal.

## Structural commentary   

**Figure 1:**
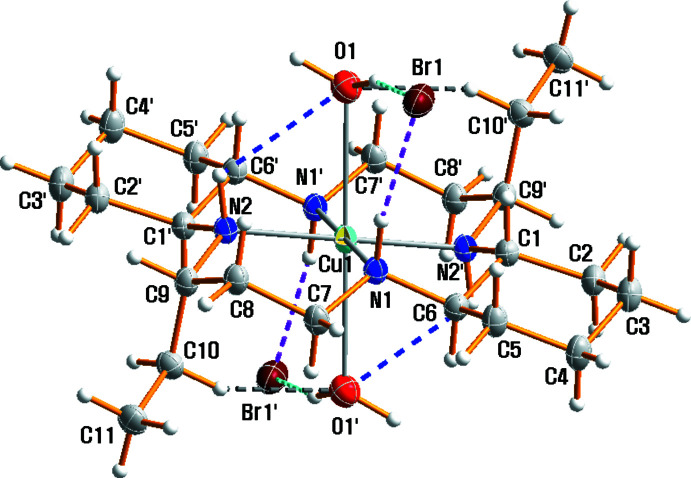
Mol­ecular structure of the [Cu1(*L*)(H_2_O)_2_]Br_2_ moiety in (I), drawn with displacement ellipsoids at the 20% probability level. Dashed lines represent hydrogen bonding inter­actions and primed atoms are related by the symmetry operation (−*x*, −*y* + 1, −*z* + 1).

Two Cu^II^ complex cations lie across a crystallographic inversion center and hence the asymmetric unit contains one half of the [Cu1(*L*)(H_2_O)_2_]^2+^ cation, one half of the [Cu2(*L*)]^2+^ cation, two bromide anions and one water solvent mol­ecule. The structures of the mol­ecular [Cu1(*L*)(H_2_O)_2_]Br_2_ and [Cu2(*L*)]Br_2_·2H_2_O moieties in (I) along with the atom-numbering scheme are shown in Figs. 1 ▸ and 2 ▸, respectively.

The macrocyclic skeletons adopt the most stable *trans*-III configuration. The Cu—N bond lengths range from 2.016 (3) to 2.055 (3) Å and are within the expected range. They are comparable to those observed in related complexes, *e.g.*, [Cu(*L*)(ClO_4_)_2_] [2.0164 (18)–2.0403 (18) Å; Lim *et al.*, 2006 ▸], [Cu(*L*)(NO_3_)_2_] [2.021 (2)–2.046 (2) Å; Choi *et al.*, 2012 ▸], [Cu(*L*)(H_2_O)_2_](SCN)_2_ [2.014 (2)–2.047 (2) Å; Choi *et al.*, 2012 ▸] and [Cu(*L*)(H_2_O)_2_]Cl_2_·4H_2_O [2.0240 (11)–2.0441 (3) Å; Moon & Choi, 2021*b* ▸]. The environments of the Cu^II^ cations may be considered as square-planar and tetra­gonally distorted octa­hedral, depending upon whether or not the out-of-plane oxygen atoms of the water mol­ecules are considered to be bonded to the copper cation. Inter­estingly, the Cu1^II^ atom exists in a tetra­gonally distorted octa­hedral environment with four nitro­gen atoms from the macrocyclic ligand in the equatorial plane and an elongated axial Cu1—O1 [2.658 (4) Å] bond owing to the Jahn–Teller distortion of *d* ^9^ copper(II) (Murphy & Hathaway, 2003 ▸) whereas the Cu2^II^ atom exists in a square-planar environment with four nitro­gen atoms from the macrocyclic ligand. The axial Cu1—O1 distance of 2.658 (4) Å in the [Cu1(*L*)(H_2_O)_2_]Br_2_ moiety is shorter than corresponding bond lengths in [Cu(*L*)(H_2_O)_2_]Cl_2_·4H_2_O [2.7866 (16) Å; Moon & Choi, 2021*b* ▸], [Cu(*L*)(ClO_4_)_2_] [2.762 (2) Å; Lim *et al.*, 2006 ▸], but it is longer than the distances in [Cu(*L*)(NO_3_)_2_] (2.506 (2) Å) or [Cu(*L*)(H_2_O)_2_](SCN)_2_ [2.569 (2) Å; Choi *et al.*, 2012 ▸]. The two ethyl groups on the six-membered chelate rings and the two –(CH_2_)_4_– parts of the cyclo­hexane backbones in (I) are *anti* with respect to the macrocyclic plane. The five-membered chelate rings adopt a *gauche* conformation and the six-membered rings are in chair conformations. The cyclo­hexane rings are also in a chair conformation, with the N atoms in equatorial positions.

## Supra­molecular features   

**Figure 3:**
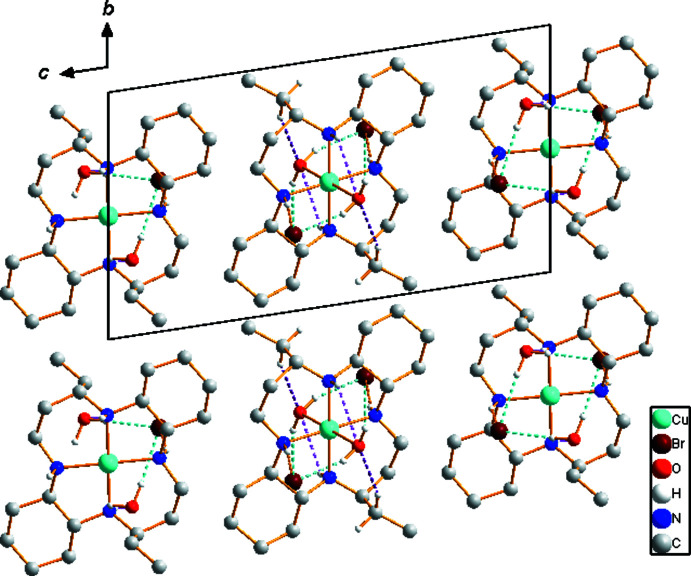
Crystal packing in (I), viewed perpendicular to the *bc* plane. Dashed lines represent O—H⋯Br (cyan), N—H⋯Br (orange), N—H⋯O (pink), and C—H⋯O (violet) hydrogen-bonding inter­actions, respectively. C-bound H atoms have been omitted.

Extensive hydrogen-bonding inter­actions occur in the crystal structure of (I); numerical details are given in Table 1 ▸. The supra­molecular architecture involves hydrogen-bonding inter­actions involving the N—H or C—H groups of the macrocycle and O—H groups of the water mol­ecules as donors, and the bromide anions as well as the O atoms of the water mol­ecules as acceptors, resulting in a chain structure extending parallel to [100] (Fig. 3 ▸). The bromide anions remain outside the coordination sphere [Cu1⋯Br1 = 4.627 (2) Å and Cu2⋯Br2 = 3.887 (3) Å] and are hydrogen-bonded to the semi-coordinating and solvent water mol­ecules through O—H⋯Br hydrogen bonds. The water solvent mol­ecule also remains outside the coordination sphere of Cu2 [Cu2⋯O2 = 4.993 (5) Å].

## Database survey   

A search of the Cambridge Structural Database (Version 5.42, update 1, Feb 2021; Groom *et al.*, 2016 ▸) indicated 19 hits for organic and transition-metal compounds containing the macrocycle (*L*, C_22_H_44_N_4_). The crystal structures of (*L*)·NaClO_4_ (Aree *et al.*, 2018 ▸), [H_2_ *L*](ClO_4_)_2_ (Aree *et al.*, 2018 ▸), [H_2_ *L*]Cl_2_·4H_2_O (Moon *et al.*, 2013 ▸), [H_2_ *L*](NO_3_)_2_·2H_2_O (Moon *et al.*, 2019 ▸), [H_4_ *L*]Cl_4_·4H_2_O (Moon & Choi, 2021*a* ▸), [H_4_ *L*]Br_4_·4H_2_O (Moon *et al.*, 2021 ▸), [H_4_ *L*](ClO_4_)_4_·2H_2_O (Moon *et al.*, 2021 ▸), [Ni(*L*)(N_3_)_2_] (Lim *et al.*, 2015 ▸), [Ni(*L*)(NCS)_2_] (Lim & Choi, 2017 ▸), {[Ni(*L*)]_0.34_[H_2_ *L*]_0.66_}Cl_2_·2H_2_O (Moon *et al.*, 2020 ▸) [Cu(*L*)(ClO_4_)_2_] (Lim *et al.*, 2006 ▸), [Cu(*L*)(NO_3_)_2_] (Choi *et al.*, 2012 ▸), [Cu(*L*)(H_2_O)_2_](SCN)_2_ (Choi *et al.*, 2012 ▸) and [Cu(*L*)(H_2_O)_2_]Cl_2_·4H_2_O (Moon & Choi, 2021*b* ▸) have been determined.

## Synthesis and crystallization   

Ethyl vinyl ketone (97%), *trans*-1,2-cyclo­hexa­nedi­amine (99%) and copper(II) bromide (99%) were purchased from Sigma-Aldrich and were used as received. All other chemicals were of analytical reagent grade. 3,14-Diethyl-2,6,13,17-tetra­aza­tri­cyclo­(16.4.0.0^7,12^)docosane (*L*) was prepared according to a published procedure (Lim *et al.*, 2006 ▸). A solution of the macrocycle *L* (0.184 g, 0.5 mmol) in 10 mL of water was added dropwise to a stirred solution of CuBr_2_ (0.113 g, 0.5 mmol) in 10 mL of water. The resulting solution was heated in a water bath for 1 h under stirring at 373 K. After cooling to 298 K, the pH was adjusted to 3.0 by the addition of 1.0 *M* HBr. The solution mixture was filtered. The filtrate was slowly evap­orated at room temperature to yield octa­hedron-like purple crystals of (I) suitable for X-ray structural analysis.

## Refinement   

Crystal data, data collection and structure refinement details are summarized in Table 2 ▸. All C- and N-bound H atoms in the complex were placed in geometrically idealized positions and constrained to ride on their parent atoms, with C—H distances of 0.97–0.99 Å, and with an N—H distance of 0.99 Å with *U*_iso_(H) values of 1.2 and 1.5*U*_eq_, respectively, of the parent atom. The hydrogen atoms of water mol­ecules were assigned based on a difference-Fourier map, and were restrained using DFIX and DANG commands during the least-squares refinement and with *U*_iso_(H) values of 1.2*U*_eq_ of the oxygen atom.

## Supplementary Material

Crystal structure: contains datablock(s) I. DOI: 10.1107/S205698902100551X/wm5611sup1.cif

Structure factors: contains datablock(s) I. DOI: 10.1107/S205698902100551X/wm5611Isup2.hkl

CCDC reference: 2086077

Additional supporting information:  crystallographic information; 3D view; checkCIF report

## Figures and Tables

**Figure 2:**
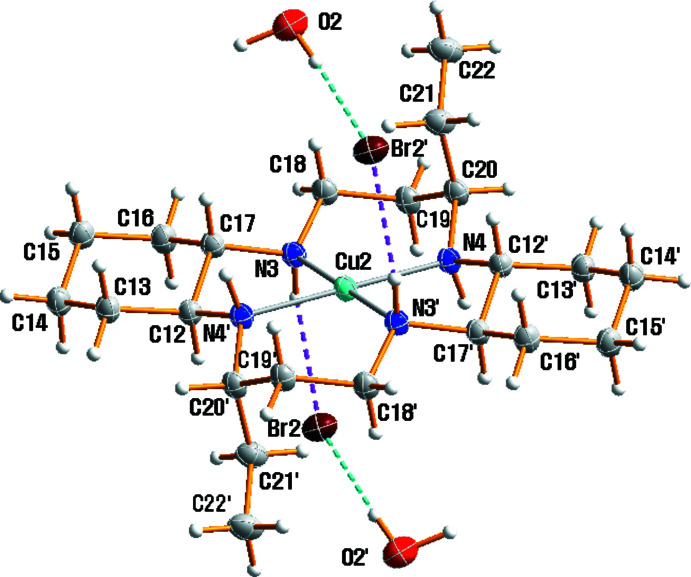
Mol­ecular structure of the [Cu2(*L*)]Br_2_·2H_2_O moiety in (I), drawn with displacement ellipsoids at the 20% probability level. Dashed lines represent hydrogen bonding inter­actions and primed atoms are related by the symmetry operation (−*x* + 1, −*y* + 1, −*z* + 2).

**Table 1:** Hydrogen-bond geometry (Å, °)

*D*—H⋯*A*	*D*—H	H⋯*A*	*D*⋯*A*	*D*—H⋯*A*
O1—H1*O*1⋯Br1^i^	0.96 (1)	2.38 (2)	3.299 (3)	160 (5)
O1—H2*O*1⋯Br1	0.96 (1)	2.39 (2)	3.314 (3)	161 (5)
O2—H1*O*2⋯Br2^ii^	0.96 (1)	2.35 (1)	3.311 (4)	176 (6)
O2—H2*O*2⋯Br2^iii^	0.96 (1)	2.38 (2)	3.335 (4)	170 (6)
N1—H1⋯Br1	0.99	2.54	3.517 (3)	171
N2—H2⋯O1	0.99	2.59	3.161 (4)	116
N3—H3⋯Br2	0.99	2.44	3.373 (3)	157
N4—H4⋯O2^iv^	0.99	1.98	2.957 (5)	169
C10—H10*A*⋯O1^v^	0.98	2.47	3.378 (5)	154

Symmetry codes: (i) -x+1, -y+1, -z+1; (ii) -x+1, -y+1, -z+2; (iii) x+1, y, z; (iv) x-1, y, z; (v) -x, -y+1, -z+1.

**Table 2:** Experimental details

Crystal data	
Chemical formula	[Cu(C_22_H_44_N_4_)(H_2_O)_2_][Cu(C_22_H_44_N_4_)]Br_4_·2H_2_O
*M*_r_	1248.00
Crystal system, space group	Triclinic, *P*\overline{1}
Temperature (K)	220
*a*, *b*, *c* (Å)	8.0800 (16), 10.380 (2), 17.511 (4)
α, β, γ (°)	97.02 (3), 92.91 (3), 111.31 (3)
*V* (Å^3^)	1350.8 (5)
*Z*	1
Radiation type	Synchrotron, λ = 0.610 Å
μ (mm^−1^)	2.53
Crystal size (mm)	0.08 × 0.07 × 0.07

Data collection	
Diffractometer	Rayonix MX225HS CCD area detector
Absorption correction	Empirical (using intensity measurements) (*HKL3000sm *SCALEPACK**; Otwinowski *et al.*, 2003 ▸)
*T*_min_, *T*_max_	0.748, 1.000
No. of measured, independent and observed [*I* > 2σ(*I*)] reflections	14201, 7233, 5311
*R*_int_	0.023
(sin θ/λ)_max_ (Å^−1^)	0.693

Refinement	
*R*[*F* ^2^ > 2σ(*F* ^2^)], *wR*(*F* ^2^), *S*	0.056, 0.176, 1.11
No. of reflections	7233
No. of parameters	298
No. of restraints	6
H-atom treatment	H atoms treated by a mixture of independent and constrained refinement
Δρ_max_, Δρ_min_ (e Å^−3^)	0.83, −1.03

Computer programs: *PAL BL2D-SMDC* (Shin *et al.*, 2016 ▸), *HKL3000sm* (Otwinowski *et al.*, 2003 ▸), *SHELXT2018* (Sheldrick, 2015*a* ▸), *SHELXL2018* (Sheldrick, 2015*b* ▸), *DIAMOND 4* (Putz & Brandenburg, 2014 ▸) and *publCIF* (Westrip, 2010 ▸).

## supplementary crystallographic information

### Crystal data

**Table:**

[Cu(C_22_H_44_N_4_)(H_2_O)_2_][Cu(C_22_H_44_N_4_)]Br_4_·2H_2_O	*Z* = 1
*M**_r_* = 1248.00	*F*(000) = 646
Triclinic, *P*1	*D*_x_ = 1.534 Mg m^−^^3^
*a* = 8.0800 (16) Å	Synchrotron radiation, λ = 0.610 Å
*b* = 10.380 (2) Å	Cell parameters from 74723 reflections
*c* = 17.511 (4) Å	θ = 0.4–33.7°
α = 97.02 (3)°	µ = 2.53 mm^−^^1^
β = 92.91 (3)°	*T* = 220 K
γ = 111.31 (3)°	Octahedron, purple
*V* = 1350.8 (5) Å^3^	0.08 × 0.07 × 0.07 mm

### Data collection

**Table:**

Rayonix MX225HS CCD area detector diffractometer	5311 reflections with *I* > 2σ(*I*)
Radiation source: PLSII 2D bending magnet	*R*_int_ = 0.023
ω scan	θ_max_ = 25.0°, θ_min_ = 1.8°
Absorption correction: empirical (using intensity measurements) (*HKL3000sm Scalepack*; Otwinowski *et al.*, 2003)	*h* = −11→11
*T*_min_ = 0.748, *T*_max_ = 1.000	*k* = −14→14
14201 measured reflections	*l* = −24→24
7233 independent reflections	

### Refinement

**Table:**

Refinement on *F*^2^	Hydrogen site location: mixed
Least-squares matrix: full	H atoms treated by a mixture of independent and constrained refinement
*R*[*F*^2^ > 2σ(*F*^2^)] = 0.056	*w* = 1/[σ^2^(*F*_o_^2^) + (0.0966*P*)^2^ + 0.5261*P*] where *P* = (*F*_o_^2^ + 2*F*_c_^2^)/3
*wR*(*F*^2^) = 0.176	(Δ/σ)_max_ = 0.001
*S* = 1.11	Δρ_max_ = 0.83 e Å^−^^3^
7233 reflections	Δρ_min_ = −1.03 e Å^−^^3^
298 parameters	Extinction correction: SHELXL-2018/3 (Sheldrick 2018), Fc^*^=kFc[1+0.001xFc^2^λ^3^/sin(2θ)]^-1/4^
6 restraints	Extinction coefficient: 0.013 (2)

### Special details

**Table:**

Geometry. All esds (except the esd in the dihedral angle between two l.s. planes) are estimated using the full covariance matrix. The cell esds are taken into account individually in the estimation of esds in distances, angles and torsion angles; correlations between esds in cell parameters are only used when they are defined by crystal symmetry. An approximate (isotropic) treatment of cell esds is used for estimating esds involving l.s. planes.

### Fractional atomic coordinates and isotropic or equivalent isotropic displacement parameters (Å^2^)

**Table:**

	*x*	*y*	*z*	*U*_iso_*/*U*_eq_	
Cu1	0.000000	0.500000	0.500000	0.04817 (16)	
Br1	0.40255 (6)	0.30972 (5)	0.58152 (3)	0.08044 (18)	
O1	0.2393 (4)	0.4149 (3)	0.43417 (19)	0.0710 (7)	
H1O1	0.345 (4)	0.481 (5)	0.418 (3)	0.107*	
H2O1	0.279 (6)	0.364 (5)	0.468 (3)	0.107*	
N1	0.0907 (4)	0.4722 (3)	0.60369 (15)	0.0481 (6)	
H1	0.186408	0.435706	0.594109	0.058*	
N2	0.1920 (4)	0.6952 (3)	0.50070 (15)	0.0469 (6)	
H2	0.294565	0.675493	0.481196	0.056*	
C1	−0.1334 (4)	0.2406 (3)	0.56209 (19)	0.0479 (6)	
H1A	−0.035569	0.211837	0.544820	0.057*	
C2	−0.2814 (5)	0.1125 (4)	0.5827 (2)	0.0555 (8)	
H2A	−0.386524	0.135435	0.592531	0.067*	
H2B	−0.315628	0.036372	0.538885	0.067*	
C3	−0.2204 (6)	0.0640 (4)	0.6541 (2)	0.0637 (9)	
H3A	−0.123680	0.031181	0.642501	0.076*	
H3B	−0.319937	−0.014341	0.668053	0.076*	
C4	−0.1551 (6)	0.1834 (4)	0.7222 (2)	0.0656 (10)	
H4A	−0.252788	0.214278	0.734951	0.079*	
H4B	−0.117270	0.150539	0.767590	0.079*	
C5	0.0004 (5)	0.3053 (4)	0.7018 (2)	0.0584 (8)	
H5A	0.099526	0.275150	0.690634	0.070*	
H5B	0.041854	0.380993	0.745878	0.070*	
C6	−0.0565 (4)	0.3587 (4)	0.63125 (19)	0.0493 (7)	
H6	−0.150780	0.394678	0.644934	0.059*	
C7	0.1707 (5)	0.5996 (4)	0.66206 (19)	0.0556 (8)	
H7A	0.214645	0.575159	0.709233	0.067*	
H7B	0.078680	0.636960	0.675206	0.067*	
C8	0.3238 (5)	0.7114 (4)	0.6327 (2)	0.0545 (8)	
H8A	0.399793	0.666842	0.608126	0.065*	
H8B	0.395973	0.778503	0.677232	0.065*	
C9	0.2703 (5)	0.7921 (4)	0.57514 (19)	0.0515 (7)	
H9	0.381547	0.865674	0.564382	0.062*	
C10	0.1486 (5)	0.8650 (4)	0.6047 (2)	0.0571 (8)	
H10A	0.032181	0.794226	0.610545	0.069*	
H10B	0.129807	0.920869	0.566305	0.069*	
C11	0.2237 (6)	0.9604 (4)	0.6822 (2)	0.0664 (10)	
H11A	0.145410	1.009367	0.696510	0.100*	
H11B	0.341660	1.027751	0.677616	0.100*	
H11C	0.231823	0.904385	0.721720	0.100*	
Cu2	0.500000	0.500000	1.000000	0.04751 (16)	
Br2	0.16473 (6)	0.61172 (5)	0.88399 (3)	0.07704 (17)	
O2	0.9627 (5)	0.3069 (4)	0.9460 (2)	0.0878 (10)	
H1O2	0.919 (9)	0.327 (6)	0.994 (2)	0.132*	
H2O2	1.007 (9)	0.394 (3)	0.925 (3)	0.132*	
N3	0.4976 (4)	0.4883 (3)	0.88410 (16)	0.0497 (6)	
H3	0.399068	0.517512	0.867864	0.060*	
N4	0.3160 (4)	0.3051 (3)	0.99739 (15)	0.0487 (6)	
H4	0.199482	0.313754	0.986331	0.058*	
C12	0.6851 (5)	0.7311 (4)	0.92254 (19)	0.0496 (7)	
H12	0.580177	0.756481	0.911901	0.060*	
C13	0.8522 (5)	0.8535 (4)	0.9107 (2)	0.0584 (8)	
H13A	0.958363	0.832878	0.923957	0.070*	
H13B	0.859883	0.937737	0.945190	0.070*	
C14	0.8480 (6)	0.8801 (4)	0.8269 (2)	0.0627 (9)	
H14A	0.749448	0.910964	0.815617	0.075*	
H14B	0.959670	0.955208	0.819902	0.075*	
C15	0.8242 (6)	0.7492 (4)	0.7708 (2)	0.0627 (9)	
H15A	0.814854	0.768115	0.717548	0.075*	
H15B	0.929007	0.724031	0.778255	0.075*	
C16	0.6574 (5)	0.6279 (4)	0.7832 (2)	0.0604 (9)	
H16A	0.647929	0.543645	0.748224	0.072*	
H16B	0.551606	0.649503	0.770759	0.072*	
C17	0.6630 (5)	0.6001 (4)	0.8667 (2)	0.0508 (7)	
H17	0.766123	0.572793	0.877565	0.061*	
C18	0.4599 (5)	0.3512 (4)	0.8348 (2)	0.0574 (8)	
H18A	0.563868	0.324513	0.840387	0.069*	
H18B	0.439538	0.360527	0.780421	0.069*	
C19	0.2978 (5)	0.2377 (4)	0.8567 (2)	0.0560 (8)	
H19A	0.265388	0.154862	0.817165	0.067*	
H19B	0.197839	0.269899	0.855880	0.067*	
C20	0.3200 (5)	0.1945 (4)	0.9351 (2)	0.0540 (7)	
H20	0.215684	0.108375	0.937994	0.065*	
C21	0.4868 (6)	0.1613 (5)	0.9475 (2)	0.0662 (10)	
H21A	0.489639	0.129926	0.997909	0.079*	
H21B	0.592161	0.247111	0.948683	0.079*	
C22	0.4980 (8)	0.0487 (5)	0.8849 (3)	0.0800 (13)	
H22A	0.603614	0.028795	0.897506	0.120*	
H22B	0.504835	0.081991	0.835259	0.120*	
H22C	0.392525	−0.035806	0.882202	0.120*	

### Atomic displacement parameters (Å^2^)

**Table:**

	*U*^11^	*U*^22^	*U*^33^	*U*^12^	*U*^13^	*U*^23^
Cu1	0.0540 (3)	0.0392 (3)	0.0440 (3)	0.0098 (2)	0.0002 (2)	0.0051 (2)
Br1	0.0674 (3)	0.0817 (3)	0.1076 (4)	0.0363 (2)	0.0237 (2)	0.0386 (3)
O1	0.0671 (16)	0.0672 (19)	0.0746 (19)	0.0222 (15)	0.0014 (13)	0.0065 (14)
N1	0.0503 (14)	0.0427 (15)	0.0453 (13)	0.0111 (12)	0.0022 (10)	0.0053 (11)
N2	0.0497 (13)	0.0396 (14)	0.0464 (14)	0.0116 (11)	0.0012 (10)	0.0053 (10)
C1	0.0501 (16)	0.0405 (17)	0.0506 (17)	0.0138 (13)	0.0032 (12)	0.0084 (12)
C2	0.0532 (18)	0.0449 (19)	0.063 (2)	0.0113 (15)	0.0034 (14)	0.0128 (15)
C3	0.062 (2)	0.051 (2)	0.070 (2)	0.0087 (17)	−0.0014 (17)	0.0190 (17)
C4	0.074 (2)	0.055 (2)	0.057 (2)	0.0088 (19)	−0.0002 (17)	0.0183 (17)
C5	0.067 (2)	0.049 (2)	0.0513 (18)	0.0111 (17)	−0.0024 (15)	0.0115 (15)
C6	0.0504 (16)	0.0464 (18)	0.0465 (16)	0.0129 (14)	0.0033 (12)	0.0073 (13)
C7	0.066 (2)	0.0471 (19)	0.0436 (16)	0.0117 (16)	−0.0014 (14)	0.0041 (13)
C8	0.0544 (17)	0.048 (2)	0.0526 (18)	0.0101 (15)	−0.0027 (13)	0.0065 (14)
C9	0.0511 (16)	0.0436 (18)	0.0508 (17)	0.0083 (14)	0.0006 (13)	0.0055 (13)
C10	0.063 (2)	0.051 (2)	0.0533 (19)	0.0195 (17)	0.0014 (14)	0.0007 (15)
C11	0.073 (2)	0.059 (2)	0.056 (2)	0.0163 (19)	0.0005 (17)	−0.0066 (17)
Cu2	0.0512 (3)	0.0412 (3)	0.0449 (3)	0.0123 (2)	0.0036 (2)	0.0032 (2)
Br2	0.0710 (3)	0.0929 (4)	0.0707 (3)	0.0396 (3)	0.00245 (19)	−0.0016 (2)
O2	0.078 (2)	0.106 (3)	0.083 (2)	0.040 (2)	0.0122 (16)	0.0070 (19)
N3	0.0540 (14)	0.0435 (15)	0.0465 (14)	0.0139 (12)	0.0027 (11)	0.0031 (11)
N4	0.0523 (14)	0.0417 (15)	0.0467 (14)	0.0123 (12)	0.0025 (11)	0.0033 (11)
C12	0.0549 (17)	0.0449 (18)	0.0460 (16)	0.0148 (14)	0.0070 (12)	0.0069 (13)
C13	0.0596 (19)	0.049 (2)	0.059 (2)	0.0106 (16)	0.0079 (15)	0.0075 (15)
C14	0.074 (2)	0.054 (2)	0.058 (2)	0.0171 (18)	0.0160 (17)	0.0136 (16)
C15	0.074 (2)	0.058 (2)	0.0533 (19)	0.0194 (19)	0.0157 (16)	0.0099 (16)
C16	0.072 (2)	0.056 (2)	0.0469 (18)	0.0171 (18)	0.0079 (15)	0.0062 (15)
C17	0.0557 (17)	0.0433 (18)	0.0497 (17)	0.0148 (15)	0.0050 (13)	0.0051 (13)
C18	0.068 (2)	0.048 (2)	0.0477 (17)	0.0136 (17)	0.0071 (14)	0.0002 (14)
C19	0.063 (2)	0.047 (2)	0.0482 (17)	0.0113 (16)	0.0007 (14)	0.0005 (14)
C20	0.0619 (19)	0.0423 (18)	0.0491 (17)	0.0120 (15)	0.0019 (14)	−0.0002 (13)
C21	0.084 (3)	0.065 (2)	0.056 (2)	0.038 (2)	0.0053 (18)	0.0025 (17)
C22	0.114 (4)	0.076 (3)	0.066 (3)	0.055 (3)	0.014 (2)	0.005 (2)

### Geometric parameters (Å, º)

**Table:**

Cu1—N1^i^	2.016 (3)	Cu2—N3	2.017 (3)
Cu1—N1	2.016 (3)	Cu2—N3^ii^	2.017 (3)
Cu1—N2^i^	2.055 (3)	Cu2—N4	2.023 (3)
Cu1—N2	2.055 (3)	Cu2—N4^ii^	2.023 (3)
Cu1—O1	2.658 (4)	O2—H1O2	0.961 (10)
O1—H1O1	0.958 (10)	O2—H2O2	0.963 (10)
O1—H2O1	0.960 (10)	N3—C18	1.490 (4)
N1—C7	1.481 (4)	N3—C17	1.492 (4)
N1—C6	1.488 (4)	N3—H3	0.9900
N1—H1	0.9900	N4—C20	1.494 (4)
N2—C1^i^	1.494 (4)	N4—C12^ii^	1.495 (4)
N2—C9	1.498 (4)	N4—H4	0.9900
N2—H2	0.9900	C12—C17	1.523 (5)
C1—C2	1.528 (5)	C12—C13	1.526 (5)
C1—C6	1.537 (5)	C12—H12	0.9900
C1—H1A	0.9900	C13—C14	1.526 (5)
C2—C3	1.528 (5)	C13—H13A	0.9800
C2—H2A	0.9800	C13—H13B	0.9800
C2—H2B	0.9800	C14—C15	1.522 (6)
C3—C4	1.528 (6)	C14—H14A	0.9800
C3—H3A	0.9800	C14—H14B	0.9800
C3—H3B	0.9800	C15—C16	1.521 (5)
C4—C5	1.518 (5)	C15—H15A	0.9800
C4—H4A	0.9800	C15—H15B	0.9800
C4—H4B	0.9800	C16—C17	1.526 (5)
C5—C6	1.530 (5)	C16—H16A	0.9800
C5—H5A	0.9800	C16—H16B	0.9800
C5—H5B	0.9800	C17—H17	0.9900
C6—H6	0.9900	C18—C19	1.514 (5)
C7—C8	1.519 (5)	C18—H18A	0.9800
C7—H7A	0.9800	C18—H18B	0.9800
C7—H7B	0.9800	C19—C20	1.516 (5)
C8—C9	1.525 (5)	C19—H19A	0.9800
C8—H8A	0.9800	C19—H19B	0.9800
C8—H8B	0.9800	C20—C21	1.520 (6)
C9—C10	1.519 (5)	C20—H20	0.9900
C9—H9	0.9900	C21—C22	1.533 (6)
C10—C11	1.531 (5)	C21—H21A	0.9800
C10—H10A	0.9800	C21—H21B	0.9800
C10—H10B	0.9800	C22—H22A	0.9700
C11—H11A	0.9700	C22—H22B	0.9700
C11—H11B	0.9700	C22—H22C	0.9700
C11—H11C	0.9700		
			
N1^i^—Cu1—N1	180.00 (7)	N3—Cu2—N3^ii^	180.0
N1^i^—Cu1—N2^i^	95.47 (11)	N3—Cu2—N4	95.18 (12)
N1—Cu1—N2^i^	84.53 (11)	N3^ii^—Cu2—N4	84.82 (12)
N1^i^—Cu1—N2	84.53 (11)	N3—Cu2—N4^ii^	84.82 (12)
N1—Cu1—N2	95.47 (11)	N3^ii^—Cu2—N4^ii^	95.18 (12)
N2^i^—Cu1—N2	180.0	N4—Cu2—N4^ii^	180.0
H1O1—O1—H2O1	106 (2)	H1O2—O2—H2O2	106 (2)
C7—N1—C6	113.3 (3)	C18—N3—C17	112.5 (3)
C7—N1—Cu1	116.0 (2)	C18—N3—Cu2	120.2 (2)
C6—N1—Cu1	107.52 (19)	C17—N3—Cu2	108.2 (2)
C7—N1—H1	106.5	C18—N3—H3	104.8
C6—N1—H1	106.5	C17—N3—H3	104.8
Cu1—N1—H1	106.5	Cu2—N3—H3	104.8
C1^i^—N2—C9	115.1 (3)	C20—N4—C12^ii^	115.3 (3)
C1^i^—N2—Cu1	107.68 (19)	C20—N4—Cu2	117.0 (2)
C9—N2—Cu1	120.8 (2)	C12^ii^—N4—Cu2	108.3 (2)
C1^i^—N2—H2	103.7	C20—N4—H4	105.0
C9—N2—H2	103.7	C12^ii^—N4—H4	105.0
Cu1—N2—H2	103.7	Cu2—N4—H4	105.0
N2^i^—C1—C2	113.8 (3)	N4^ii^—C12—C17	107.6 (3)
N2^i^—C1—C6	105.7 (3)	N4^ii^—C12—C13	113.6 (3)
C2—C1—C6	112.5 (3)	C17—C12—C13	111.0 (3)
N2^i^—C1—H1A	108.2	N4^ii^—C12—H12	108.1
C2—C1—H1A	108.2	C17—C12—H12	108.1
C6—C1—H1A	108.2	C13—C12—H12	108.1
C3—C2—C1	111.2 (3)	C12—C13—C14	110.7 (3)
C3—C2—H2A	109.4	C12—C13—H13A	109.5
C1—C2—H2A	109.4	C14—C13—H13A	109.5
C3—C2—H2B	109.4	C12—C13—H13B	109.5
C1—C2—H2B	109.4	C14—C13—H13B	109.5
H2A—C2—H2B	108.0	H13A—C13—H13B	108.1
C2—C3—C4	110.6 (3)	C15—C14—C13	111.5 (3)
C2—C3—H3A	109.5	C15—C14—H14A	109.3
C4—C3—H3A	109.5	C13—C14—H14A	109.3
C2—C3—H3B	109.5	C15—C14—H14B	109.3
C4—C3—H3B	109.5	C13—C14—H14B	109.3
H3A—C3—H3B	108.1	H14A—C14—H14B	108.0
C5—C4—C3	110.2 (3)	C16—C15—C14	110.8 (3)
C5—C4—H4A	109.6	C16—C15—H15A	109.5
C3—C4—H4A	109.6	C14—C15—H15A	109.5
C5—C4—H4B	109.6	C16—C15—H15B	109.5
C3—C4—H4B	109.6	C14—C15—H15B	109.5
H4A—C4—H4B	108.1	H15A—C15—H15B	108.1
C4—C5—C6	110.4 (3)	C15—C16—C17	111.0 (3)
C4—C5—H5A	109.6	C15—C16—H16A	109.4
C6—C5—H5A	109.6	C17—C16—H16A	109.4
C4—C5—H5B	109.6	C15—C16—H16B	109.4
C6—C5—H5B	109.6	C17—C16—H16B	109.4
H5A—C5—H5B	108.1	H16A—C16—H16B	108.0
N1—C6—C5	114.2 (3)	N3—C17—C12	106.1 (3)
N1—C6—C1	106.3 (3)	N3—C17—C16	113.7 (3)
C5—C6—C1	111.3 (3)	C12—C17—C16	110.6 (3)
N1—C6—H6	108.3	N3—C17—H17	108.7
C5—C6—H6	108.3	C12—C17—H17	108.7
C1—C6—H6	108.3	C16—C17—H17	108.7
N1—C7—C8	111.7 (3)	N3—C18—C19	111.5 (3)
N1—C7—H7A	109.3	N3—C18—H18A	109.3
C8—C7—H7A	109.3	C19—C18—H18A	109.3
N1—C7—H7B	109.3	N3—C18—H18B	109.3
C8—C7—H7B	109.3	C19—C18—H18B	109.3
H7A—C7—H7B	107.9	H18A—C18—H18B	108.0
C7—C8—C9	115.8 (3)	C18—C19—C20	115.6 (3)
C7—C8—H8A	108.3	C18—C19—H19A	108.4
C9—C8—H8A	108.3	C20—C19—H19A	108.4
C7—C8—H8B	108.3	C18—C19—H19B	108.4
C9—C8—H8B	108.3	C20—C19—H19B	108.4
H8A—C8—H8B	107.4	H19A—C19—H19B	107.4
N2—C9—C10	112.3 (3)	N4—C20—C19	109.5 (3)
N2—C9—C8	108.6 (3)	N4—C20—C21	111.4 (3)
C10—C9—C8	114.4 (3)	C19—C20—C21	113.2 (3)
N2—C9—H9	107.1	N4—C20—H20	107.5
C10—C9—H9	107.1	C19—C20—H20	107.5
C8—C9—H9	107.1	C21—C20—H20	107.5
C9—C10—C11	112.9 (3)	C20—C21—C22	113.5 (4)
C9—C10—H10A	109.0	C20—C21—H21A	108.9
C11—C10—H10A	109.0	C22—C21—H21A	108.9
C9—C10—H10B	109.0	C20—C21—H21B	108.9
C11—C10—H10B	109.0	C22—C21—H21B	108.9
H10A—C10—H10B	107.8	H21A—C21—H21B	107.7
C10—C11—H11A	109.5	C21—C22—H22A	109.5
C10—C11—H11B	109.5	C21—C22—H22B	109.5
H11A—C11—H11B	109.5	H22A—C22—H22B	109.5
C10—C11—H11C	109.5	C21—C22—H22C	109.5
H11A—C11—H11C	109.5	H22A—C22—H22C	109.5
H11B—C11—H11C	109.5	H22B—C22—H22C	109.5
			
N2^i^—C1—C2—C3	−172.3 (3)	N4^ii^—C12—C13—C14	177.4 (3)
C6—C1—C2—C3	−52.1 (4)	C17—C12—C13—C14	56.0 (4)
C1—C2—C3—C4	55.5 (5)	C12—C13—C14—C15	−55.6 (5)
C2—C3—C4—C5	−59.7 (5)	C13—C14—C15—C16	55.8 (5)
C3—C4—C5—C6	59.7 (5)	C14—C15—C16—C17	−56.3 (5)
C7—N1—C6—C5	−61.5 (4)	C18—N3—C17—C12	−178.7 (3)
Cu1—N1—C6—C5	169.1 (3)	Cu2—N3—C17—C12	−43.5 (3)
C7—N1—C6—C1	175.5 (3)	C18—N3—C17—C16	59.5 (4)
Cu1—N1—C6—C1	46.0 (3)	Cu2—N3—C17—C16	−165.4 (3)
C4—C5—C6—N1	−176.1 (3)	N4^ii^—C12—C17—N3	54.6 (3)
C4—C5—C6—C1	−55.7 (4)	C13—C12—C17—N3	179.5 (3)
N2^i^—C1—C6—N1	−58.0 (3)	N4^ii^—C12—C17—C16	178.3 (3)
C2—C1—C6—N1	177.2 (3)	C13—C12—C17—C16	−56.7 (4)
N2^i^—C1—C6—C5	177.1 (3)	C15—C16—C17—N3	176.2 (3)
C2—C1—C6—C5	52.3 (4)	C15—C16—C17—C12	56.9 (4)
C6—N1—C7—C8	178.4 (3)	C17—N3—C18—C19	176.7 (3)
Cu1—N1—C7—C8	−56.6 (3)	Cu2—N3—C18—C19	47.4 (4)
N1—C7—C8—C9	76.6 (4)	N3—C18—C19—C20	−68.4 (4)
C1^i^—N2—C9—C10	52.8 (4)	C12^ii^—N4—C20—C19	173.1 (3)
Cu1—N2—C9—C10	−79.2 (3)	Cu2—N4—C20—C19	−57.8 (3)
C1^i^—N2—C9—C8	−179.8 (3)	C12^ii^—N4—C20—C21	−60.9 (4)
Cu1—N2—C9—C8	48.3 (3)	Cu2—N4—C20—C21	68.2 (4)
C7—C8—C9—N2	−69.3 (4)	C18—C19—C20—N4	74.7 (4)
C7—C8—C9—C10	57.0 (4)	C18—C19—C20—C21	−50.3 (4)
N2—C9—C10—C11	177.9 (3)	N4—C20—C21—C22	−179.8 (4)
C8—C9—C10—C11	53.6 (4)	C19—C20—C21—C22	−55.8 (5)

Symmetry codes: (i) −*x*, −*y*+1, −*z*+1; (ii) −*x*+1, −*y*+1, −*z*+2.

### Hydrogen-bond geometry (Å, º)

**Table:**

*D*—H···*A*	*D*—H	H···*A*	*D*···*A*	*D*—H···*A*
O1—H1*O*1···Br1^iii^	0.96 (1)	2.38 (2)	3.299 (3)	160 (5)
O1—H2*O*1···Br1	0.96 (1)	2.39 (2)	3.314 (3)	161 (5)
O2—H1*O*2···Br2^ii^	0.96 (1)	2.35 (1)	3.311 (4)	176 (6)
O2—H2*O*2···Br2^iv^	0.96 (1)	2.38 (2)	3.335 (4)	170 (6)
N1—H1···Br1	0.99	2.54	3.517 (3)	171
N2—H2···O1	0.99	2.59	3.161 (4)	116
N3—H3···Br2	0.99	2.44	3.373 (3)	157
N4—H4···O2^v^	0.99	1.98	2.957 (5)	169
C10—H10*A*···O1^i^	0.98	2.47	3.378 (5)	154

Symmetry codes: (i) −*x*, −*y*+1, −*z*+1; (ii) −*x*+1, −*y*+1, −*z*+2; (iii) −*x*+1, −*y*+1, −*z*+1; (iv) *x*+1, *y*, *z*; (v) *x*−1, *y*, *z*.
